# Designing and integrating a quality management program for patients undergoing head and neck resection with free-flap reconstruction

**DOI:** 10.1186/s40463-020-00436-3

**Published:** 2020-06-23

**Authors:** Joseph C. Dort, Khara M. Sauro, Christiaan Schrag, Shamir Chandarana, Jennifer Matthews, Steven Nakoneshny, Vida Manoloto, Tanya Miller, C. David McKenzie, Robert D. Hart, T. Wayne Matthews

**Affiliations:** 1 Section of Otolaryngology Head & Neck Surgery, Department of Surgery, University of Calgary Cumming School of Medicine, 3280 Hospital Drive NW, Calgary, Alberta T2N 4Z6 Canada; 2 Ohlson Research Initiative, Arnie Charbonneau Research Institute, University of Calgary Cumming School of Medicine, Calgary, Canada; 3 Foothills Medical Centre, Alberta Health Services, Calgary, Alberta Canada; 4 Department of Community Health Sciences, University of Calgary Cumming School of Medicine, Calgary, Canada; 5 Section of Plastic and Reconstructive Surgery, Department of Surgery, University of Calgary Cumming School of Medicine, Calgary, Canada

**Keywords:** Head and neck cancer, Care pathways, Clinical pathways, Head and neck surgery, Clinical outcomes improvement, Quality improvement, Healthcare delivery

## Abstract

**Background:**

Care pathways (CPs) offer a proven method of systematically improving patient care. CPs are particularly helpful in complex clinical conditions where variation in care is a problem such as patients undergoing major head and neck resection with free flap reconstruction. Although CPs have been used to manage this patient group, most CPs are implemented as part of relatively short-term quality improvement projects. This paper outlines a detailed methodology for designing and delivering a quality management program sustained for 9 years.

**Methods:**

We describe a change management approach informed by Kotter’s “8 Step Process” that provided a useful framework to guide program development and implementation. We then provide a detailed, step by step description of how such a program can be implemented as well as a detailed summary of time and costs for design, implementation and sustainability phases. An approach to design and delivery of a measurement, audit and feedback system is also provided.

**Results:**

We present a summary of resources needed to design and implement a head and neck surgery quality management program. The primary result of this study is a design for a sustainable quality management program that can be used to guide and improve care for patients undergoing major head and neck resection with free flap reconstruction.

**Conclusions:**

A change management approach to design and delivery of a head and neck quality management program is practical and feasible.

## Introduction and background

Head and neck cancers are the 7th most common malignancies worldwide and surgery is a mainstay of treatment. In many cases, major procedures involving resection and reconstruction of critical head and neck structures are necessary. Major surgery is complex and time-consuming with overall procedure times that routinely take 8–12 h, particularly when free flap reconstruction is required [1]. Complications after major head and neck procedures are common and may include wound infection [2], flap compromise [3, 4], and pneumonia [5], any of which can lead to significant delay in healing and recovery. Postoperative hospital length of stay (LOS) is often 14 days or longer [6]. Although modern head and neck surgery regularly achieves success, its complexity and cost have led providers to seek better ways to design and deliver care.

Quality management approaches have been used since the 1990’s and have been shown to improve the quality of care. High quality care is safe, effective, efficient, equitable, timely and patient-centered [7]. Quality management often focuses on using tools, such as care pathways, to enable providers to organize and deliver high-quality evidence-based care. Care pathways are complex interventions that facilitate decision-making with regards to care for a defined patient population [8]. Care pathways have been shown to improve survival in other conditions, such as severe Adult Respiratory Distress Syndrome [9–11] . Care pathways have also been developed and integrated for patients undergoing major head and neck surgery - Cohen and colleagues reported the first study where a care pathway was used for these patients [12]. Since that time there have been numerous studies of care pathway utilization in the management of head and neck cancer and most have shown significant improvements in clinical and financial outcomes [5, 6, 13–17].

Most studies reporting on the use of care pathways for head and neck cancer report the results of quality improvement projects, as opposed to quality management programs. A quality improvement project, when well designed, has a project charter that includes a defined scope, budget and a finite project duration. Projects therefore have a beginning, a middle and an end. When a project concludes, the resources – people, funding and attention – allocated to that project are typically reassigned to other projects. Unfortunately, the usual consequence is that the improved clinical outcomes achieved during a project are not maintained and often clinical performance returns to its pre-project level. Not only is this wasteful, it is also demoralizing and sends the wrong message to patients and front-line care providers. In order to sustain high clinical performance, quality improvement must be sustainable and extended over time [18]. Conversely, quality management programs enable not only short-term quality improvement but also support sustained, ongoing quality control (continuous quality management).

Although the head and neck cancer care pathway work highlighted earlier has achieved improved clinical outcomes, none of the current literature describes a method for designing, integrating and sustaining a quality management program for patients undergoing major head and neck resection with free flap reconstruction. In this paper we present a step-by-step method to develop a quality management program designed, integrated and sustained at a tertiary, academic head and neck surgery program in Calgary, Alberta, Canada. We also provide data illustrating the time and resources required for each step of the design process. The methods described herein, however, can be applied to clinical programs seeking to improve their processes of care and clinical outcomes.

## Program setting

The head and neck surgery clinical outcomes assessment program (the Calgary Program) was formally established at the Foothills Medical Centre (FMC) in Calgary, Alberta, Canada in 2012 after 2 years of development. FMC is the largest academic teaching hospital in Alberta, serving a population of approximately 2 million people in the Southern half of the province. Over 300 head and neck surgical procedures are performed per year at FMC with over 50 of these involving major resection with free flap reconstruction. FMC is a publicly-funded institution operating in a single-payer provincial healthcare system. The Ohlson Research Initiative is a clinical translational research program focused on healthcare quality management as well as the design and delivery of high value healthcare. The pathway development work described in this manuscript was conducted with support and guidance from the Ohlson Research Initiative.

## Resources

The program resources shown in Table 1 are estimates of time and costs during the different phases of the program. Boundaries between different phases are not fixed and project phases can, and do, overlap. Resources required for ongoing, post-integration maintenance are summarized in Table 2. Program sustainability is commonly overlooked, therefore understanding the ongoing costs of maintaining a program is critically important and is therefore included here.

**Table 1:** Program Development Resources

Domains	Role	Responsibility	Hours / Week	Estimated Cost (including benefits)	Comment
**Change Management** **Clinical Design Team** **Pathway Development** **Computerized Order Entry**	Program Lead / Champion	Leads and coordinates the overall program, articulates the need for change, answers questions. Guides data collection and analysis and also development of measurement, audit and feedback system.	5	Variable funding depends on local circumstances.	The program lead and clinical champion are ideally, but not necessarily, the same individual. This individual should be a respected member of the team and should have the requisite leadership skills to enable successful program design, implementation and maintenance.
	Project Coordinator	Coordinates project work, meetings, etc.	2	$40 / hr	This role was performed by the Calgary program lead. This may not be transferable to other programs.
	QI Consultant	Ensures QI methodologies and measurement are being used appropriately.	4	$50 / hr	This role was performed by the Calgary program lead. This may not be transferable to other programs.
	Data Collection	Chart abstraction and data entry for baseline cohort.	250	$20 / hr	This is a one time expense for baseline data collection. This will vary depending on centre.
	Analyst	Cleans, validates and analyses clinical data. Develops, along with clinical lead, the measurement audit and feedback system as well as the minimum data set.	5	$52 / hr	Total analytic hours during program development were 250.
	Clinicians - MD	Shares knowledge of clinical processes, helps with data interpretation, participates in creating new processes.	1	Variable funding depends on local circumstances.	Different clinicians were more or less involved at different phases. Goal is to minimize clinician time needed for meetings. Some jursdictions compensate physicians for time spent in quality management program development.
	Clinicians - RN	Shares knowledge of clinical processes, helps with data interpretation, participates in creating new processes.	1	$50 / hr	Different clinicians were more or less involved at different phases. Goal is to minimize clinician time needed for meetings.
	Clinicans - Allied Health	Shares knowledge of clinical processes, helps with data interpretation, participates in creating new processes.	1	$50 / hr	Different clinicians were more or less involved at different phases. Goal is to minimize clinician time needed for meetings.
	Information Techology (IT) Consultant	Works with team to translate clinical pathway / protocol into the hospital electronic medical record. Liaison with order set developers with IT.	4	$50 / hr	This individual works with the team on an "as-needed" basis. Total hours required are less than 50. Once order sets are developed the IT Consultant is available to implement pathway modifications.

**Table 2:** Program Maintenance Resources

Domains	Role	Responsibility	Hours / Week	Estimated Cost	Comment
**Program Guidance Council** **Pathway Maintenance** **Computerized Order Entry**	Program Lead / Champion	Leads and coordinates the overall program, articulates the need for change, answers questions. Guides data collection and analysis and also development of measurement, audit and feedback system.	3	Variable funding depends on local circumstances.	The program lead's responsibilities are similar to those during the design phase. Fewer hours are required but ongoing focus and leadership is critically important.
	Administrative Assistant	Coordinates team meetings	0.5	$45 / hr	Administrative support to assist the program lead with meetings is a valuable support.
	QI Consultant	Ensures QI methodologies and measurement are being used appropriately.	1	$50 / hr	This role was performed by the Calgary program lead. This may not be transferable to other programs.
	Data Collection	Ongoing collection of key performance indicators at point of care.	3.5	$45 / hr	Current program uses some manual data collection. A new hospital EMR might should reduce the need for manual collection.
	Analyst	Cleans, validates and analyses clinical data. Maintains, along with clinical lead, the measurement audit and feedback system as well as the minimum data set. Prepares and distributes quality management reports.	5	$52 / hr	Data analysis and reporting are ongoing program costs and represent a major cost of sustainability.
	Clinicians - MD	Shares knowledge of clinical processes, helps with data interpretation, participates in creating new processes.	0.5	Variable funding depends on local circumstances.	MDs participate in program guidance council meetings and can become more involved depending on interest and availability.
	Clinicians - RN	Shares knowledge of clinical processes, helps with data interpretation, participates in creating new processes.	0.5	$50 / hr	RN leaders from the inpatient unit attend program guidance council meetings and also assist with training of unit nursing staff.
	Clinicans - Allied Health	Shares knowledge of clinical processes, helps with data interpretation, participates in creating new processes.	0.5	$50 / hr	Allied health leaders from the inpatient unit attend program guidance council meetings and also assist with communication among allied health staff.
	Information Techology (IT) Consultant	Works with team to translate clinical pathway / protocol into the hospital electronic medical record. Liaison with order set developers with IT.	0.5	$50 / hr	This individual works with the team on an "as-needed" basis. Total hours required are less than 50. Once order sets are developed the IT Consultant is available to implement pathway modifications.

Overall leadership for program design, development and integration was provided by the lead author (JCD) who has expertise in quality management, health system design / implementation and the development of measurement, audit and feedback systems for quality improvement.

It took 2 years to design and fully implement the head and neck care pathway including creation of a measurement, audit and feedback system. Details of the Calgary Program development are described here.

## Change management – a guiding principle

**Fig. 1:**
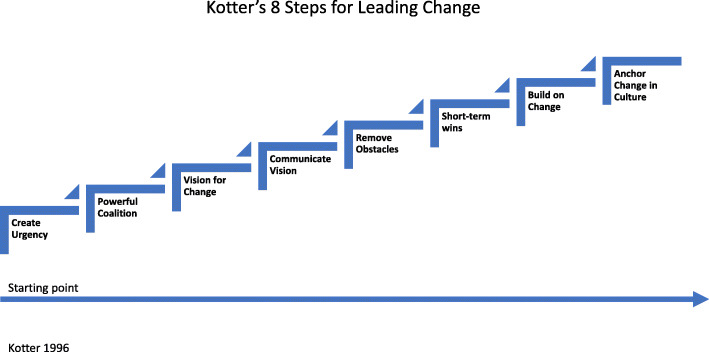
Kotter’s 8 Steps for Leading Change

Designing and implementing a quality management program presents a major challenge to existing workflows, practice patterns, team dynamics and team culture. Managing and adapting to these challenges requires a change management framework. The “8 step process” articulated by Kotter [19] (see Fig. 1) provided a useful guide to managing change in the Calgary Program. The first step in Kotter’s framework is to create a sense of urgency. In Calgary this was accomplished by collecting and analyzing data from 2005 to 2009 that showed high rates of pulmonary complications, readmissions to ICU and prolonged length of stay in the resection with free flap patient group [5]. These data, when presented to the team, created an urgent need to improve clinical performance. The clinical design team was formed (as outlined below) and work to create the guiding coalition, shape the vision and communicate it to all stakeholders began. The team was empowered, and resourced, to make the changes required to improve clinical performance. The contemporaneous development of a measurement, and audit and feedback system enabled data to be reported back to the team and provided powerful evidence of early gains in clinical performance. Allocating dedicated, stable resources to the quality management program and reinforcing the new way of managing clinical care resulted in a shift in culture that promotes evidence-based and quality-focused patient care. New workflows and processes along with a dedicated measurement and audit and feedback system have become the “new normal”. These changes form the foundation for sustainable clinical improvement.

## Forming a team: Clinical Design Team & Clinician Engagement

Designing and integrating a quality management program requires a team. Members of the clinical design team included: surgeons, physicians, nurses, allied health professionals, trainees (residents), a quality improvement expert, a data management / analytics expert and additional members as required. Patients were not formally engaged in the initial pathway design but were involved in refining some aspects of the program (see Patient Engagement below).

We leveraged committed leadership of clinical champions who are respected by their peers to develop and sustain the pathway. Outcomes data showing a gap between current outcomes and expected outcomes were a powerful motivator for ongoing development and maintenance of the pathway. A clinical champion with quality management expertise (JCD) led these discussions, which were often challenging, but ultimately resulted in a shared understanding and agreement about care processes that would be implemented in a care pathway.

Once the initial design was completed and integrated the design team then became formally responsible for ongoing refinement of the protocols including management of the minimum data set, reports and other program activities. These steps ensure ongoing team engagement because the team is responsible and empowered to make changes. As the program transitions from design and integration into sustainability the clinical design team becomes a program guidance council that is focused on reviewing results, identifying problems and opportunities and making changes to the care pathway. Many of the same people are involved but the time required is less intense. Ongoing engagement is enabled through regular clinical management meetings, circulation of quality management reports and empowering the team to make changes to the pathway based on outcomes data and evolving practice standards. Resources required for program sustainability are shown in Table 2.

## Pathway development

As noted above, the clinical design team was responsible for developing the pathway. Pathway development took place in three interrelated and iterative stages over a period of approximately 8 months: 1) process mapping of current clinical process, 2) literature review to identify “best practices” to inform the care pathway and 3) prioritization process to identify essential components of the care pathway. As work progressed, smaller working groups refined specific clinical processes using their clinical judgement supplemented by the literature review. Evolving drafts were circulated to team members and poster versions of the evolving pathway were placed in report rooms on the inpatient unit. Staff members were invited to “mark-up” the posters with their comments and suggestions. Marked-up posters were then taken down and suggestions were incorporated into a revised pathway that was then re-posted for further feedback. This lengthy, iterative process was an innovative and pragmatic approach chosen to avoid the need for multiple large meetings with busy design team members. It also allowed all staff members to reflect and comment at their leisure in a relaxed environment. As time progressed fewer comments were added and the design team then finalized the first draft that was ready for patient testing.

**Fig. 2:**
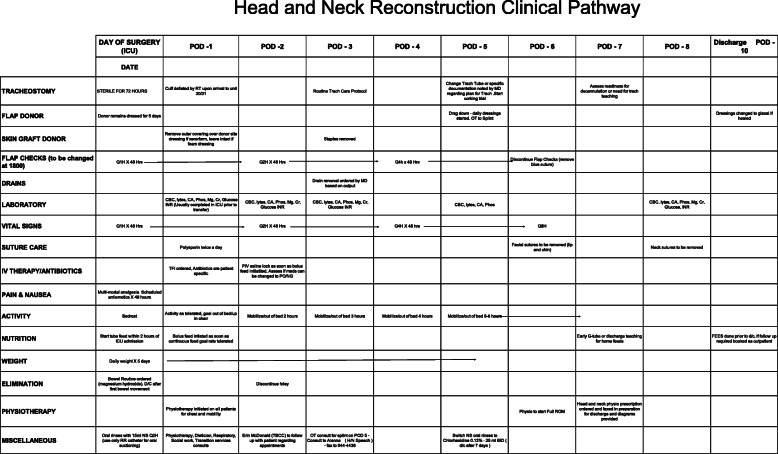
The Calgary Care Pathway

Care providers from all disciplines were actively engaged in providing feedback and the care pathway was further refined until the present 10-day pathway was finalized. The current version of the pathway is shown in Fig. 2. The pathway continues to evolve to incorporate new evidence and to respond to the needs of patients. Changes to the care pathway are managed by the clinical design team and updated in the hospital EMR via Computerized Order Entry (COE; see below).

## Removing barriers: Pathway Integration & Computerized Order Entry

The care pathway was initially integrated in early 2010 as a paper-based process. After several months of testing and refining, work began on designing the computerized order sets and was fully integrated in the hospital EMR in early 2011.

The Foothills Medical Centre deployed a hospital EMR in 2007 (Sunrise Clinical Manager, Allscripts Corp, Chicago, USA). The system is designed for COE, results management and clinical documentation. There is also embedded decision support. The EMR system allows creation of complex, multi-item order sets, whereby specific components of order set can be triggered on specified dates and times as specified by the care pathway. This capability was important during the transition from paper-based to computer-enabled care pathway integration. Development of computerized order sets was complex and took about 6 months to design and integrate. The clinical design team was engaged in order set development and testing through meetings with the technical development group. The critically important technical support for this process was provided by our provincial health authority. Important and detailed end-user testing was conducted by using the order set to deliver real time clinical care and noting any shortcomings. End-user feedback was collected and used to revise the order sets. Once computerized order sets were deployed, delivery of care that was compliant with the care pathway became faster and more reliable.

The care pathway is initiated by a member of the surgical team, either a staff surgeon or surgical resident, usually at the end of a major procedure. The care pathway order set can be initiated with a single click and therefore evidence-informed “best care” becomes the default. While a standard set of orders are automatically pre-selected in the EMR when the care pathway order set is chosen, the care pathway is flexible and responsive to individual patient needs. The care pathway can be customized by simply deselecting standard orders.

The clinical design team maintains an ongoing active role in monitoring the care pathway, a process that is enabled by a dedicated measurement system that includes audit and feedback of key clinical outcomes (explored below in Measurement & Evaluation). This ongoing measurement and audit and feedback system is a critically important component of the quality management program.

In addition to integration within the EMR there were also face-to-face training sessions with all members of the team. New staff members continue to be oriented to care pathway processes and the COE system. Training sessions include residents and students working with the clinical team.

## Measurement & Evaluation

### Defining a minimum data set

A minimum data set defines the vital few variables necessary for quality management. The process of defining a minimum data set was undertaken very early in the program’s development and took approximately 6 months. This process of defining the minimum data set involved multiple (approximately 6) meetings with all clinical design team members in order to reach consensus. During this phase there was vigorous discussion about which outcomes should be addressed in the care pathway as well as what should and should not be measured. There was frequent tension between two goals, namely collecting data for its intended purpose versus being comprehensive. As the number of variables tracked increases, so does the cost and complexity of data collection. Therefore, the overall design objective was to define a parsimonious set of relevant measures that could be readily collected without disrupting clinical workflow. Where possible, measures routinely collected in the hospital EMR were used. Other data elements are collected manually using the methods and processes described below.

A parsimonious set of eight key performance indicators (KPIs) created as a result of this process. The KPIs include days to: unit arrival, mobilization, tube feeds started, Foley catheter removed, tracheotomy tube downsized, tracheotomy decannulation, 2-day decannulation delay and length of stay. Because of the beneficial impact timely decannulation has on pulmonary complications, three of the KPIs are focused on tracheotomy management [5].

Complications are also an important part of the quality management program and for this reason 10 important complications are routinely tracked: pneumonia, return to OR, flap compromise, flap loss, return to ICU, pulmonary embolism, deep vein thrombosis, myocardial infarction, delirium and death.

### Data collection and analysis

Data collection and analysis are critically important foundations to all quality management programs; we cannot improve what we cannot measure. Therefore, considerable time and attention were paid to the design of the measurement and analysis system. Clinical data, as defined by the minimum data set, are prospectively collected by a trained research assistant who is embedded on the hospital inpatient unit. Clinical data are obtained while the patient is an inpatient through a combination of paper chart review and data abstraction from the EMR. Information pertaining to complications are recorded in the chart by the attending physician and / or resident physicians. Charts are reviewed for sentinel events, including: a return to the OR, the ICU or prescription of a new medication that is not part of the standard clinical pathway. Physician, nursing and allied health professional notes are reviewed to obtain information on important pathway milestones (e.g., mobilization time, removal of drains and catheters or removal of a tracheotomy tube). Diagnostic imaging reports are also reviewed to supplement paper chart and EMR data.

### Data reporting

The goal of a reporting system is to provide timely, informative and actionable outputs that pertain to the clinical program of interest. Reports are tailored to the target end-users. They are brief and make abundant use of graphics and simple tables that quickly and efficiently display outcomes. Program team meetings are held bi-monthly and attended by physicians, nurses, allied health professionals, unit managers and the data collection and analysis team. The data report is prioritized and as such is the first agenda item discussed. The outcomes are used to modify current care protocols. In this manner an iterative cycle of continuous quality improvement can be used to inform ongoing clinical care. The report is also circulated to key hospital administrators to keep leadership attention focused on program performance, outcomes and improvements.

## Patient engagement

Patient involvement in clinical design is an evolving process and is recognized as a relative weakness of this program. The patient perspective was initially obtained during the development phase using paper notes. Careful notes were kept about what worked well and what did not. Patients also attend clinical design team meetings; however, this was on an ad hoc basis rather than a regular part of the meeting.

Patients are introduced to the pathway during the preoperative assessment clinic visit. A care pathway progress chart is placed in each patient room and patients and family members are encouraged to frequently refer to this chart and use it to understand how their recovery is progressing, which milestones are being met, and which still need to be addressed. The care pathway progress chart also enables patients and family members to engage in and ask questions about their care. Patients tend to be more motivated to recover when they can clearly see that they are meeting important milestones. The chart therefore serves as a touch point for communication with patients and families. Quality outcomes and care pathway highlights are also displayed in outpatient clinic examination rooms and patient feedback is sought on how patients experienced the processes of care.

## Discussion

In this paper we outline a practical approach to designing and integrating a quality management program for patients undergoing major head and neck surgery with free flap reconstruction. We also provide a detailed description of the time and resources needed to design, integrate and maintain a head and neck quality management program.

There has been an increased interest in the integration of care pathways into the care of patients undergoing head and neck oncologic procedures [13, 15, 16, 20–23]. The appeal of care pathways is supported by improvements in processes of care and clinical outcomes [5, 6, 17, 24, 25]. Our program has demonstrated sustained high clinical performance when compared to a non-pathway managed cohort using 7 years of prospective outcomes data. Similarly, we have previously shown that hospital length of stay (LOS), an important measure of overall clinical performance, is consistently lower in the Calgary Program care pathway patients and there is no evidence that shorter LOS has an adverse impact on readmission rates or emergency department visits [17]. Other authors have also demonstrated successful improvement in clinical outcomes through the use of care pathways [12] [14, 15].

Despite the list of studies demonstrating improved outcomes from integrating care pathways for head and neck surgical patients, long-term outcomes from formal quality management programs are lacking. All the cited studies report results from short-term projects usually lasting from 1 to 3 years. Furthermore, none of the current studies describe the steps required to design and integrate a quality management program. By reporting long-term results in our companion paper as well as details on the design and integration of the Calgary Program we hope to facilitate the uptake of care pathways in other centres. Understanding the resource implications of starting and sustaining a quality management program is also important. In this paper we have provided data outlining the time and costs required to design, integrate and maintain a head and neck surgery quality management program.

One of the strengths of the Calgary Program care pathway is that it is an ongoing and evolving care pathway deployed within a learning healthcare system. This allows it to be responsive to changes in clinical outcomes as well as new evidence. For example, recent work using an enhanced recovery after surgery (ERAS) protocol for patients undergoing resection with free flap reconstruction, which have been found to reduce hospital LOS [26] to be integrated. Conversely, the current care pathway reported in this study has some significant gaps. There are no data reported on pain control, management of postoperative nausea and vomiting, prehabilitation or quantification of mobilization after surgery. These important gaps are being closed with recent pathway modifications and we anticipate improvements in future outcomes. Stable support for the quality management program is an important enabler of continuous pathway improvement.

## Conclusions

By thoroughly describing a systematic approach to designing, integrating and sustaining a quality management program for patients undergoing major head and neck resection with free flap reconstruction, including resources required for such a program, we hope to encourage the design and integration of care pathways in other centres. We have also highlighted some of the strengths and limitations of the care pathway. We believe that this approach is an important strategy to maintaining excellent clinical performance in complex and resource-challenged healthcare environments.

## Data Availability

The data that support the findings of this study are available from the authors but restrictions apply to the availability of these data, which contain identifiable information, for the current study, and so are not publicly available. Data are however available from the authors upon reasonable request and with permission of the office of the privacy commissioner of Alberta.
